# Synthesising conceptual frameworks for patient and public involvement in research – a critical appraisal of a meta-narrative review

**DOI:** 10.1186/s12874-018-0572-0

**Published:** 2018-10-25

**Authors:** David Evans, Noreen Hopewell-Kelly, Michele Kok, Jo White

**Affiliations:** Department of Health and Social Sciences, University of the West of England, Frenchay Campus, Bristol, BS16 1QY UK

**Keywords:** Conceptual framework, Critical appraisal, Patient and public involvement, Meta-narrative review, Synthesis

## Abstract

**Background:**

A number of conceptual frameworks for patient and public involvement (PPI) in research have been published in recent years. Although some are based on empirical research and/or existing theory, in many cases the basis of the conceptual frameworks is not evident. In 2015 a systematic review was published by a collaborative review group reporting a meta-narrative approach to synthesise a conceptual framework for PPI in research (hereafter ‘the synthesis’). As the first such synthesis it is important to critically scrutinise this meta-narrative review. The ‘RAMESES publication standards for meta-narrative reviews’ provide a framework for critically appraising published meta-narrative reviews such as this synthesis, although we recognise that these were published concurrently. Thus the primary objective of this research was to appraise this synthesis of conceptual frameworks for PPI in research in order to inform future conceptualisation.

**Methods:**

Four researchers critically appraised the synthesis using the RAMESES publication standards as a framework for assessment. Data were extracted independently using a data extraction form closely based on the RAMESES publication standards. Each item from the standards was assessed on a four point scale (0 = unmet, 1 = minimally met, 2 = partly met, 3 = fully met). The four critical appraisals were then compared and any differences resolved through discussion.

**Results:**

A good degree of inter-rater reliability was found. A consensus assessment of the synthesis as a meta-narrative review of PPI conceptual frameworks was achieved with an average of ‘1’ (minimally met) across all 20 items. Two key items (‘evidence of adherence to guiding principles of meta-narrative review’ and ‘analysis and synthesis processes’) were both wholly unmet. Therefore the paper did not meet our minimum requirements for a meta-narrative review. We found the RAMESES publication standards were a useful tool for carrying out a critical appraisal although some minor improvements are suggested.

**Conclusions:**

Although the aims of the authors’ synthesis were commendable, and the conceptual framework presented was coherent and attractive, the paper did not demonstrate a transparent and replicable meta-narrative review approach. There is a continuing need for a more rigorous synthesis of conceptual frameworks for PPI.

## Background

A number of conceptual frameworks for patient and public involvement (PPI) in health services or research have been published in recent years [1–5]. Although some are based on empirical research and/or existing theory, in many cases the basis of the conceptual frameworks is not evident; in addition, they use very different and inconsistent terminology to describe their work including “conceptual frameworks” [1], “models” [5], “taxonomies” [6], and “typologies” [7]. Other papers refer to “values” [8], “principles” [9], “measures” [10] or “standards” [11] of PPI yet these papers also offer some degree of conceptualising PPI. Some of these frameworks refer to and build on previous conceptualisations, but many fail to refer to or build on existing work. Systematic reviews of PPI often refer to the regrettable lack of consistent terminology and adequate conceptualisation in the field. A systematic review by Brett and colleagues which specifically looked at the conceptualisation of PPI concluded:

“Those papers that have focused on conceptualisation are often based on reflection or opinion rather than more formal conceptual development or theoretical development or testing, which has not yet occurred in the field of PPI” [12].

It is not even clear what ‘counts’ as a conceptual framework, for example whether those articles focusing on values, principles, measures or standards for PPI represent conceptual frameworks.

Brett and colleagues identified the challenges in this area but did not attempt a synthesis of conceptual frameworks for PPI. Thus it is of great interest that a recent systematic review has been published by a collaborative review group reporting the use of a meta-narrative approach to synthesise a framework for PPI in research [13]. They report:

“Utilizing a systematic review, environmental scan and manual search of peer-reviewed literature and other sources regarding PSUE [patient and service user engagement – their term for PPI] in biomedical and health services research, this paper synthesizes a standardized, evidence-based framework for understanding, reporting and assessing PSUE to jump-start a reliable and comparative evidence base” [13].

Two hundred and two sources met their inclusion criteria for the systematic review and they report that 41 of these presented some framework or conceptualisation of PSUE. These 41 papers were then synthesised into a two-part framework for PSUE. Four essential components were identified: patient and service user initiation, building reciprocal relationships, co-learning, and re-assessment and feedback. Additionally, three broad phases of PSUE in research (preparatory, execution and translational) were described, each comprising more detailed specific stages. As the first and currently sole synthesis of conceptual frameworks for PPI in research it is important to critically assess this meta-narrative systematic review, particularly as the article is now regularly cited in the PPI literature.

As part of the evidence-based movement, the last two decades have seen the development of a network of reporting guidelines including CONSORT for randomised controlled trials, PRISMA for systematic reviews and most recently GRIPP2 for PPI in research [14]. A key underlying principle of all these approaches is that any published evidence synthesis should be transparent in its methodology and replicable by any other researchers with access to the raw data. The ‘RAMESES publication standards for meta-narrative reviews’ [15] provides a tool which can be adapted for critically appraising published meta-narrative reviews such as this synthesis of PPI conceptual frameworks. Meta-narrative review is one of a number of new approaches to qualitative and mixed methods evidence synthesis which include meta-ethnography [16], realist synthesis [17] and critical interpretive synthesis [18]. RAMESES (Realist And MEta-narrative Evidence Syntheses) is a network of researchers and others seeking to develop and promote both realist and meta-narrative review methodologies. Meta-narrative review was first developed by Greenhalgh et al. in 2004 as a response to the challenges that emerged in a review of service-level innovations in health care [19]. It is described by the authors of the RAMESES publication standards: “A meta-narrative review seeks to illuminate a heterogeneous topic area by highlighting the contrasting and complementary ways in which researchers have studied the same or similar topic” [15]. Meta-narrative reviews are ideally suited to illuminating and clarifying complex topic areas such as PPI in health services research where terminology and concepts are unclear or contested, and where the lack of quantitative studies means traditional systematic review methods are unlikely to produce a meaningful synthesis. The RAMESES publication standards for meta-narrative reviews have been cited 129 times to date, indicating the wide interest in this method, but to our knowledge this is the first time they have been used as a tool for critical appraisal of a published meta-narrative review. As the synthesis authors state explicitly that their paper was a meta-narrative review, the RAMESES publication standards were the most appropriate tool with which to critically appraise the paper. We recognise, however, that the RAMESES standards were not published until 2013, at the same time as this synthesis paper, so the authors may not have had access to the standards when writing their paper (although some guidance was available through the RAMESES website and network before final publication of the standards). Therefore in our discussion below we also seek to contextualise their work with reference to the 2005 Greenhalgh et al. paper which the synthesis authors reference, and which introduced and provided a model for conducting a meta-narrative review [19].

Thus the primary objective of this research was to critically appraise the quality of the synthesis of conceptual frameworks for PPI in research. A secondary objective was to trial the RAMESES publication standards for meta-narrative reviews as a tool for critically appraising a published meta-narrative review. Thus, we hope this critical appraisal may serve as an exemplar to help future authors of both conceptual frameworks for PPI and meta-narrative reviews more generally.

## Methods

The authors of the RAMESES publication standards provide detailed guidance on how to use the standards, including 20 items for reporting, followed by examples and an explanation of each item and how it can be used. We created a critical appraisal data extraction template with all 20 RAMESES meta-narrative review publication standards and for each standard, two additional cells: one for the evidence of the extent to which we judged the paper under review met the standard, and one for rating the paper against the item on a four-point scale (0 = unmet, 1 = minimally met, 2 = partly met, 3 = fully met).

The critical appraisal was carried out by four members of a research team (co-authors of the current paper) with a collective focus on and experience of PPI in health services research. There is little guidance in the evidence synthesis literature on how many reviewers to include beyond the advice in the Cochrane Handbook (and elsewhere) that it is “desirable for more than one author to repeat parts of the process” [20]. We chose to involve four researchers as meta-narrative review is a relatively new methodology and experience in critical appraisal of such reviews is limited. By including four researchers, each appraising independently and initially blind to the other three assessments, we aimed to maximise the validity of our analysis and conclusions. We also sought further information from the corresponding author of the synthesis paper but did not receive a reply.

The four researchers independently appraised the synthesis paper using the data extraction template. The four appraisals were then aggregated with the evidence for each item assessment collated, and both an average and the range of ratings recorded for each item. Qualitative comments were compared and any differences resolved through discussion but logged for reporting. The number and scale of ratings were too small for a statistical analysis of inter-rater reliability to be meaningful, but we checked for inter-rater reliability with a traffic light system: green (high) where there was no more than one-score difference between any of the four assessors; amber (medium) where there was up to two scores difference between any two members of the team; red (low) where there was at least one difference of three scores between any two members of the team. Where there was a difference of either two (amber) or three (red) scores, the item was further discussed by the team and a consensus score agreed.

Although there is no indicator of a minimum standard in the RAMESES guidelines, we agreed the need to set a minimum average value across all indicators for the paper to be judged a satisfactory meta-narrative review. We agreed the value of ‘2’, equivalent to the assessment of ‘partly met’ for individual items, was an appropriate minimum average across all items for a paper to be judged satisfactory. In addition, although the RAMESES publication standards do not weight the different items, we agreed that two key indicators of acceptable quality were item 7 (evidence of adherence to the guiding principles of meta-narrative review) and item 12 (analysis and synthesis processes). Our view was that after reaching a consensus score for these two items, our assessment of both should be a minimum ‘2’ in addition for the average of all item scores to be ‘2’ in order for the paper to be judged a satisfactory meta-narrative review.

## Ethics

Secondary analysis such as ours do not normally require ethical review or consent procedures, but one of our reviewers raised the important question of whether we should have sought permission from the authors for this critical appraisal. Having discussed this as a team, we believe we were right to inform the lead/corresponding author that we were conducting a critical appraisal, but that it would have been potentially detrimental to open academic debate to ask for permission to publish, as were permission denied we would then be ethically restricted from publishing. A dialogue with the authors might well have improved the quality of our paper and helped us minimise any biases or errors in our analysis. As we did not receive a response to seeking supplementary information from the lead/corresponding author, including two further approaches, we judged it ethically and methodologically acceptable to then seek to publish independently.

## Results

The results of our critical appraisal of the synthesis paper are presented in Table 1. Column 1 states the item, column 2 presents our initial scores, column 3 the average across our four independent assessments, column 4 presents our collective consensus score after discussion and column 5 summarises the evidence to support our assessment. After discussion, the assessors were able to reach consensus on all scores. As the table demonstrates, we did not find the synthesis paper to provide a satisfactory meta-narrative review of PPI conceptual frameworks either in terms of the overall average score (average of 1 below our minimum required average score of ‘2’) or in terms of the two key indicators (items 7 and 12 both scored ‘0’ as opposed to our required minimum of ‘2’). It was notable that our highest degree of inter-rater consistency was on items 7 and 12 where in both instances all four assessors independently scored these key items as ‘0’. The average score across all items before discussion was 1.15 while after discussion this decreased slightly to 1. In either case, this was well below our minimum requirement for a satisfactory meta-narrative review.

**Table 1:** Critical appraisal of the synthesis paper as a meta-narrative review

Item	Initial scores	Ave. score	Final score	Evidence summary
Title				
1. In the title, identify the document as a meta-narrative review or synthesis.	0, 3, 0, 2	1.25	0	Called a ‘systematic review and synthesised framework’ rather than a meta-narrative review or synthesis.
Abstract				
2. While acknowledging publication requirements and house style, abstracts should ideally contain brief details of: the study’s background, review questions or objectives; search strategy; methods of selection, appraisal, analysis and synthesis of sources; main results; and implications for practice.	1, 2, 1, 3	1.75	2	Most required elements are present but no research question stated and no mention of any of the methodological approaches outlined for meta-narrative review in the RAMESES publication standards.
Introduction				
3. *Rationale for review*: explain why the review is needed and what it is likely to contribute to existing understanding of the topic area.	1, 2, 2, 3	2	2	Rationale for doing a systematic review given but no mention of meta-narrative review in the rationale.
4. *Objectives and focus of review*: state the objective(s) of the review and/or the review question(s). Define and provide a rationale for the focus of the review.	0, 2, 1, 1	1	1	The broad objective of the review is stated but the review question(s) are never explicitly stated.
Methods				
5. *Changes in the review process*: any changes made to the review process that was initially planned should be briefly described and justified.	0, 0, 0, 0	0	0	No mention made of any changes in the review process.
6. *Rationale for using meta-narrative review*: explain why meta-narrative review was considered the most appropriate method to use.	0, 1, 1, 1	0.75	1	The first mention of the employment of the meta-narrative approach is very late – under the Analysis section, and the approach is broadly referenced rather than rationalized. Inadequate mention is made of the research tradition and epistemological synthesis and critique etc., and the attempt to build an overarching narrative which characterize meta-narratives.
7. *Evidence of adherence to guiding principles of meta-narrative review*: where appropriate, show how each of the six guiding principles (pragmatism, pluralism, historicity, contestation, reflexivity and peer review) has been followed.	0, 0, 0, 0	0	0	No evidence of adherence to guiding principles of meta-narrative review and no mention of any of the guiding principles.
8. *Scoping the literature*: describe and justify the initial process of exploratory scoping of literature.	1, 2, 0, 3	1.5	2	There is evidence of an initial systematic review of the literature which can be considered a scoping exercise. This is coherent and covers a good range of databases. However, this is not identified as a scoping of the literature preliminary to a meta-narrative review.
9. *Searching processes*: while considering specific requirements of the journal or other publication outlet, state and provide a rationale for how the iterative searching was done. Provide details on all the sources accessed for information in the review. Where searching in electronic databases has taken place, the details should include (for example) name of database, search terms, dates of coverage and date last searched. If individuals familiar with the relevant literature and/or topic area were contacted, indicate how they were identified and selected.	2, 1, 1, 1	1.25	1	Even as a traditional systematic review this was imperfect, for example, there is no clear statement of the review question or search terms. Other aspects of a traditional systematic review are adequately described.
10. *Selection and appraisal of documents*: explain how judgements were made about including and excluding data from documents, and justify these.	2, 1, 1, 0	1	1	Inclusion and exclusion criteria for the systematic review are not detailed sufficiently and there is insufficient clarity on sources used. Methods for data extraction are unclear, there is little information available regarding databases used, search terms, dates of coverage and so on.
11. *Data extraction*: described and explain which data or information were extracted from the included documents and justify this selection.	2, 0, 2, 1	1.25	1	Data extraction is described but not justified. Study selection section provides very little justification. Team disagreements leading to inclusion and further scrutiny is the only example of this.
12. *Analysis and synthesis processes*: describe the analysis and synthesis processes in detail. This section should include information on the constructs analysed and describe the analytic process.	0, 0, 0, 0	0	0	A meta-narrative approach is claimed but no details are given on data analysis and synthesis. The paper does not include an exploration of different traditions and constructs, but instead appears to synthesize different components and processes related to PSUE outlined in the literature reviewed, and their inter-relations.
Results				
13. *Document flow diagram*: provide details on the number of documents assessed for eligibility and included in the review with reasons for exclusion at each stage as well as an indication of their source of origin (for example, from searching databases, reference lists and so on).	2, 1, 2, 2	1.75	2	A document flow diagram is included which shows a reduction from 202 studies in the systematic review to 41 studies included in synthesised framework. The text explains the rationale for why the 41 were included and the others excluded. Little other detail is given at this stage, for example their source of origin. Most importantly, there is no list or table of the 41 included studies so the authors’ decision-making cannot be examined or tested.
14. *Document characteristics*: provide information on the characteristics of the documents included in the review.	0, 0, 0, 2	0.5	0	No details are given on the 41 studies included in the analysis.
15. *Main findings*: Present the key findings with a specific focus on theory building and testing.	2, 2, 1, 1	1.5	1	The four components are presented clearly but where the different phases are introduced the focus on theory building and testing becomes unclear.
Discussion				
16. *Summary of findings*: summarise the main findings, taking into account the review’s objective(s), research question(s), focus and intended audience(s).	2, 2, 0, 1	1.25	1	The findings are potentially interesting as it is the first (to our knowledge) attempt to provide a synthesis of conceptual frameworks for PPI based on a comprehensive review of other frameworks; however the lack of a clear statement of the review’s objectives and research question, make it difficult to judge how well they have achieved their objectives.
17. *Strengths, limitations and future research directions*: discuss both the strengths of the review and its limitations. These should include (but need not be restricted to) (a) consideration of all the steps in the review process and (b) comment on the overall strength of evidence supporting the explanatory insights which emerged. The limitations identified may point to areas where further work is needed.	1, 1, 2, 2	1.5	1	The main strength of the review is that they have produced a conceptual framework for PPI based on a systematic review and a synthesis of 41 other conceptual papers. Many other conceptual frameworks for PPI appear to have little or no prior review of previous conceptualisations. The main weaknesses/limitations of the review are its many methodological inconsistencies and omissions, and in particular that the authors do not appear to have conducted anything that can be recognised as the meta-narrative approach they claim. Most of the stages of a meta-narrative review are either not present or only minimally present. There is not enough detail on the analysis to judge the validity of the framework of four components for PPI arrived at.
18. *Comparison with existing literature*: where applicable, compare and contrast the review’s findings with the existing literature (for example, other reviews) on the same topic.	2, 1, 1, 1	1.25	1	The ‘Comparison with other systematic reviews’ section (p.1160) carries out some of the necessary comparative critique but is tied to processes, not paradigms or research tradition analysis, and therefore does not meet the requirements of meta-narrative synthesis.
19. *Conclusion and recommendations*: list the main implications of the findings and place these in the context of other relevant literature. If appropriate, offer recommendations for policy and practice.	1, 1, 1, 1	1	1	The findings are compared with other systematic reviews but not placed in context of other conceptual frameworks. Authors claim their work provides “a framework with broad applicability and cohesive underpinnings necessary to integrate existing knowledge and guide future endeavours” but due to its significant methodological weaknesses we do not consider their framework to be robust.
20. *Funding*: provide details of funding sources (if any) for the review, the role played by the funder (if any) and any conflicts of interests of the reviewers.	3, 2, 3, 2	2.5	2	Partial funding source given (Patient-Centered Outcomes Research Institute) but no information on rest of funding.
Overall	1.15	1.15	1	This paper does not fulfil the criteria for a meta-narrative review as set out in the Rameses publication standards. Overall methodological quality is poor, and the conceptual model of PPI presented is therefore not well supported.

Source: Items in italics are based on Wong G, GreenhalghT, Westhorp G, Buckingham J, Pawson R. RAMESES publication standards: meta-narrative reviews. *BMC Medicine* 2013; 11:20

In terms of inter-rater reliability, overall there was a good degree of consistency between our four independent assessments across the 20 items. There was a high degree of inter-rater reliability in 11 items (items 5,6,7,9,12,13,15,17,18,19,20), a medium degree of consistency in seven items (items 2,3,4,10,11,14,16) and a low degree in only two items (items 1,8). For item 1, two assessors gave ‘0’ as the term ‘meta-narrative synthesis’ was not mentioned in the title; two assessors gave higher scores as the title included ‘synthesis’ and the item specified “meta-narrative review or synthesis.” The difference here was thus due to the ambiguity as to whether the ‘or’ in the item allowed a high score to a title that included ‘synthesis’ without a preceding ‘meta-narrative’. In discussion we agreed that the item meant a ‘meta-narrative synthesis’ and therefore a consensus score of ‘0’ was agreed. With item 8 the difference in assessment was due to differing judgements as to whether the first stage systematic review was a scoping exercise preliminary to a meta-narrative review as required by the item, or whether it was actually the main research method to which the claimed meta-narrative review was in fact secondary. In discussion we agreed that although not presented as a scoping exercise it could be interpreted. In each case we achieved a consensus score after discussion.

## Discussion

The aim of the authors to synthesise PPI conceptual frameworks was commendable, and constitutes an important and necessary task in a field which has too long been fraught with conceptual uncertainty. It was also a challenging enterprise, given no obvious available method for conducting such a synthesis. Their eventual synthesis into a two-part framework with four components (patient and service user initiation, building reciprocal relationships, co-learning process, and re-assessment and feedback) and three phases (preparatory, execution and translational) presents useful insights and raises interesting questions for the conceptualisation of PPI. Moreover, the authors expand the conceptual framework in some detail, and the four components, three phases and nine stages are clearly referenced to the wider PPI literature they reviewed. It was not, however our objective to make an assessment of the coherence or value of their conceptualisation, which would have required a different methodological approach from our critical appraisal of their application of the meta-narrative review methodology.

What our critical appraisal did reveal, however, were some crucial limitations in synthesis related to the lack of clarity and transparency in the methodology employed. As our appraisal demonstrates, it does not meet current standards for a meta-narrative review since a number of key aspects of that approach are missing. In particular, there is no evidence of the six guiding principles of meta-narrative review: pragmatism, pluralism, historicity, contestation, reflexivity and peer review. Given the RAMESES standards had not been finally published at the time of synthesis authors’ work, this is perhaps unsurprising (although some guidance was available through the RAMESES website and network before final publication of the standards). But there is also no reference to the six phases outlined in the earlier 2005 Greenhalgh et al. paper which the authors cite and the key emphasis already present there on identifying and analysing different research tradition narratives. Moreover, it is unknown how the authors got from the 41 articles they started with to their eventual conceptual framework; indeed the 41 articles are not tabulated or referenced, therefore making it impossible for others to judge or replicate their analysis. Overall, much more methodological detail is given for their traditional systematic review than for the meta-narrative review.

The lack of transparency and replicability in the synthesis paper is comparable with a number of other conceptual frameworks for PPI which, similarly, lack robust empirical, theoretical and/or methodological bases. Since the publication of the synthesis paper, however, several other articles have been published which put forward new conceptual frameworks for PPI, sometimes with stronger foundations. For example, Gibson, Welsman and Britten have produced a second iteration of their “four-dimensional theoretical framework” for PPI which they have now tested in empirical workshops [21]. Hamilton et al. have recently published “an empirically based conceptual framework for fostering meaningful patient engagement in research” [22]. Although not a syntheses of existing frameworks, these papers have the merit of an empirical basis in work with patients and the public, and in the case of Gibson, Welsman and Britten, both an empirical and theoretical basis.

As noted above, the synthesis paper has been cited numerous times, and its continued citations suggest it has an established status within the PPI field and offers a coherent and intuitively attractive PPI conceptual framework. Yet the lack of methodological detail provided raises the need for a more robust and transparent synthesis of PPI conceptual frameworks. This is not, however, an easy or unproblematic task. One key challenge is defining and identifying what ‘counts’ as a PPI conceptual framework. As discussed at the beginning of this article, authors use a variety of terms including ‘conceptual framework’, ‘model’, ‘theory’ and ‘theoretical framework’ to describe similar processes in the conceptualisation of PPI. Some conceptual frameworks have a wholly theoretical basis, some an empirical basis and some a combination of the two. Jabareen has helpfully provided a general definition and discussion of conceptual frameworks [23]. He defines “conceptual framework as a network, or a ‘plane,’ of interlinked concepts that together provide a comprehensive understanding of a phenomenon or phenomena.” Jabareen emphasises that building a conceptual framework is an iterative process that requires an understanding of the relationships between the concepts that provide the building blocks for the overall framework. Conceptual frameworks are indeterminate in nature and need to draw on multidisciplinary bodies of knowledge. Thus the search terms and inclusion criteria for identifying the literature for any further synthesis will need careful thought. There is then the question of which methodological approach to synthesis is most appropriate. There is a range of approaches from the traditional systematic review [24], meta-ethnography [16], realist review [25], critical interpretive synthesis [18] and of course meta-narrative review [19].

This is not the place to weigh the respective merits of these different approaches to evidence synthesis, but suffice to say that an argument can certainly be made for the appropriateness of a meta-narrative review to be repeated. If done well it has the benefits of an explicit, transparent and repeatable process. Indeed, our own critical appraisal could easily be replicated and tested by any researchers as both the original synthesis paper and the RAMESES publication standards are open access. Thus the RAMESES publication standards provide a well-developed and tested method for evidence synthesis, and a number of useful meta-narrative reviews have now been published in the field of health research [19].

We had a secondary objective to trial the RAMESES publication standards as a tool for critically appraising a published meta-narrative review, a use for which they were not initially explicitly intended. From our perspective, this trial was successful; the RAMESES publication standards could be easily and effectively adapted as a critical appraisal tool. We transferred the standards into a critical appraisal checklist and applied them with four researchers independently assessing the 20-item tool. There was a high degree of inter-rater reliability and we found it easy to apply most of the items. There was a low degree of consistency on the assessment of only two of the 20 items, and we were able to easily resolve the inconsistency and agree scores in these cases through discussion. In the cases of divergence, these were mainly caused by two key issues. First, some of the language in the RAMESES standards was ambiguous. As discussed above, the most important impact of this was on the first item, where the location of the ‘or’ in the standard led to different interpretations of whether the existence of the term ‘synthesis’ in the title was or was not sufficient to meet the standard. The second main area of divergence was in relation to a specific characteristic of the synthesis paper where the authors report both a traditional systematic review and a follow-up meta-narrative review on a sub-sample of the systematic review papers. Initially some researchers in the team applied the standards to both methods, but in discussion we agreed that the standards should only be applied to the meta-narrative review element of the paper. Once these initial differences of approach had been resolved in principle, it was straight-forward to agree a final score by consensus. There is always a risk in consensus processes that stronger voices will dominate weaker ones, so in retrospect it might have been better to have re-scored anonymously; this did not occur to us at the time, but we will do so in any future critical appraisal.

The one key change we found we needed in adapting the publication standards was to give a weighting to what we judged were the two most important items (items 7 and 12). Of course the publication standards could still be used without this weighting, if others thought this unnecessary or inappropriate. In the case of our appraisal of the synthesis paper, this would not have changed our final assessment as the paper scored weakly across all 20 items as well as the two weighted items.

In conducting this critical appraisal we were struck by how scarce the public scrutiny of the most regularly cited papers in the PPI literature is; although there have been a number of systematic reviews of the PPI literature, to our knowledge, no individual and detailed critical appraisal has been published of any of these papers. Critical appraisal is a key element of knowledge production and synthesis, and should not be restricted to the peer review process or quality appraisal within systematic reviews and other forms of evidence synthesis, important though these processes are. The peer review process can be fallible, [26] thus we need more critical appraisal tools, and more published critical appraisals within the PPI literature, particularly in the area of conceptual frameworks, where it is only through greater intellectual rigour that more coherent conceptualisations of PPI, which are clearly needed, will be realised.

Finally, it is perhaps important to note that after we had completed our critical appraisal, we became aware of a recent overview of systematic reviews of PPI in clinical trials which included the synthesis paper in its assessment [27]. Of the 27 systematic reviews included, the quality of this paper was rated second lowest and one of six in the low quality category. Other than listing it in a quality appraisal table, the overview article did not specifically discuss the synthesis paper, and its focus was on the systematic review rather than the conceptual framework, but this assessment was consistent with our conclusion that the paper lacked clarity and transparency in its methodology.

## Conclusions

It is unusual (but not unknown) to publish a critical appraisal of an individual journal paper; where it is done, it is often in the form of a letter to the journal, sometimes with space also made for a rebuttal by the original authors [28]. Such debates about the quality of research can only challenge all researchers to strive for higher level of methodological robustness and rigour. Often, such critical appraisals are done privately in journal clubs or academic study, but in undertaking our work on the synthesis paper we have come to see the importance of placing such critical appraisals in the public domain as a contribution to knowledge generation in PPI. It is widely acknowledged that there is a great need for more robust conceptualisation of PPI, and thus an equal need for more rigorous syntheses of conceptual frameworks for PPI. We were excited when we first saw this paper as it was, to our knowledge, the first attempt to produce such a synthesis. Although offering an interesting and coherent conceptual model of PPI, this synthesis paper did not in fact meet the criteria for a meta-narrative review laid down by the RAMESES publication guidelines nor did it follow the model of the earlier Greenhalgh et al. meta-narrative review it cited; important aspects of the synthesis process were missing, including crucially any details on the 41 papers the authors used in their synthesis. It was disappointing that their potentially very useful PPI conceptual framework did not provide a transparent and replicable methodology, nevertheless this critical appraisal has usefully highlighted that there is a continuing, even urgent, need for a more rigorous synthesis of conceptual frameworks for PPI. As flawed exemplars are often the most useful for learning, we hope that this critical appraisal will assist future authors to develop more robust synthesis, whether through meta-narrative review or other methodologies.

## Abbreviations

PPI: Patient and public involvement
PSUE: Patient and service user engagement
RAMESES: Realist And MEta-narrative Evidence Syntheses

## References

[1] Charles C, DeMaio S (1993). Lay participation in health care decision making: a conceptual framework. J Health Polit Policy Law.

[2] Oliver S, Rees R, Clarke-Jones L, Milne R, Oakley A, Gabbay J, Stein K, Buchanan P, Gyte G (2008). A multidimensional conceptual framework for analysing public involvement in health services research. Health Expect.

[3] Tritter J (2009). Revolution or evolution: the challenges of conceptualizing patient and public involvement in a consumerist world. Health Expect.

[4] Li K, Abelson J, Giacomini M, Contandriopoulos D (2015). Conceptualizing the use of public involvement in health policy decision-making. Soc Sci Med.

[5] Morrow E, Ross F, Grocott P, Bennett J (2010). A model and measure for quality service user involvement in health research. Int J Consum Stud.

[6] Thompson G (2007). The meaning of patient involvement and participation in health care consultations: a taxonomy. Soc Sci Med.

[7] Rowe G, Frewer L (2005). A typology of public engagement mechanisms. Sci Technol Hum Values.

[8] Gradinger F, Britten N, Wyatt K (2015). Values associated with public involvement in health and social care research: a narrative review. Health Expect.

[9] Baines Rebecca L., Regan de Bere Sam (2017). Optimizing patient and public involvement (PPI): Identifying its “essential” and “desirable” principles using a systematic review and modified Delphi methodology. Health Expectations.

[10] Abelson J, Li K, Wilson G, Shields K, Schneider C, Boesveld S (2015). 2015 Supporting quality public and patient engagement in health system organizations: development and usability testing of the public and patient engagement evaluation tool. Health Expect.

[11] INVOLVE. *Values, Principles and standards for public involvement in research.* Eastleigh: INVOLVE 2014.

[12] Brett J, Staniszewska S, Mockford C, Seers K, Herron-Marx S, Bayliss H (2015). The PIRICOM study: a systematic review of the conceptualisation, measurement, impact and outcomes of patients and public involvement in health and social care research.

[13] Shippee N, Domecq Garces J, Prutsky Lopez G (2013). Patient and service user engagement in research: a systematic review and synthesized framework. Health Expect.

[14] Staniszewska S, Brett J, Simera I (2017). GRIPP 2 reporting checklist: tools to improve reporting of patient and public involvement in research. Res Involv Engagem.

[15] Wong G, Greenhalgh T, Westhorp G, Buckingham J, Pawson R (2013). RAMESES publication standards: meta-narrative reviews. BMC Med.

[16] Britten N, Campbell R, Pope C, Donovan J, Morgan M, Pill R (2002). Using meta ethnography to synthesise qualitative research – a worked example. J Health Serv Res Policy.

[17] Pawson R, Greenhalgh T, Harvey G, Walshe K (2005). Realist review – a new method of systematic review designed for complex policy interventions. J Health Serv Res Policy.

[18] Dixon-Woods M, Cavers D, Agarwal S, Annandale E, Arthur A, Harvey J, Hsu R, Katbamna S, Olsen R, Smith L, Riley R, Sutton A (2006). Conducting a critical interpretive synthesis on the literature on access to healthcare by vulnerable groups. BMC Med Res Methodol.

[19] Greenhalgh T, Robert G, Macfarlane F (2005). Storylines of research in diffusion of innovation: a meta-narrative approach to systematic review. Soc Sci Med.

[20] Higgins J, Green S, editors. Cochrane handbook for systematic reviews of interventions Version 5.1.0 [updated March 2011] The Cochrane Collaboration Section 7.2.4. Available from www.handbook.cochrane.org.

[21] Gibson Andy, Welsman Jo, Britten Nicky (2017). Evaluating patient and public involvement in health research: from theoretical model to practical workshop. Health Expectations.

[22] Hamilton Clayon B., Hoens Alison M., Backman Catherine L., McKinnon Annette M., McQuitty Shanon, English Kelly, Li Linda C. (2017). An empirically based conceptual framework for fostering meaningful patient engagement in research. Health Expectations.

[23] Jabareen Y (2009). Building a conceptual framework: philosophy, definitions and procedure. Int J Qual Methods.

[24] Gough D, Oliver S, Thomas J (2012). An introduction to systematic reviews.

[25] Pawson R, Greenhalgh T, Harvey G, Walshe K (2004). Realist synthesis: an introduction. ESRC Research Methods Programme.

[26] Lipworth W, Kerridge I, Carter SM, Little M (2011). Journal peer review in context: a qualitative study of the social and subjective dimensions of manuscript review in biomedical publishing. Soc Sci Med.

[27] Price Amy, Albarqouni Loai, Kirkpatrick Jo, Clarke Mike, Liew Su May, Roberts Nia, Burls Amanda (2017). Patient and public involvement in the design of clinical trials: An overview of systematic reviews. Journal of Evaluation in Clinical Practice.

[28] Weidinger S, Baurecht H, Schmitt J (2015). A critical appraisal of the PETITE study report: topical corticosteroids are safe and effective in the long-term treatment of infantile atopic dermatitis. Paediatrics.

